# Divergent Requirements for Glutathione Biosynthesis During Osteoclast Differentiation In Vitro and In Vivo

**DOI:** 10.3390/antiox14020197

**Published:** 2025-02-10

**Authors:** Guoli Hu, Amy L. Whitaker, Guo-Fang Zhang, Courtney M. Karner

**Affiliations:** 1 Department of Internal Medicine, University of Texas Southwestern Medical Center, Dallas, TX 75390, USA; 2 Department of Medicine, Division of Endocrinology, Metabolism Nutrition, Duke University Medical Center, Durham, NC 27701, USA; guofang.zhang@duke.edu; 3 Sarah W. Stedman Nutrition and Metabolism Center & Duke Molecular Physiology Institute, Duke University School of Medicine, Durham, NC 27701, USA; 4 Charles and Jane Pak Center for Mineral Metabolism and Clinical Research, University of Texas Southwestern Medical Center, Dallas, TX 75390, USA

**Keywords:** glutathione, osteoclast, bone

## Abstract

Glutathione (GSH) is the most abundant antioxidant in the cell, and it is responsible for neutralizing reactive oxygen species (ROS). ROS can promote osteoclast differentiation and stimulate bone resorption and are some of the primary drivers of bone loss with aging and loss of sex steroids. Despite this, the role of GSH biosynthesis during osteoclastogenesis remains controversial. Here, we show that the requirements for GSH biosynthesis during osteoclastogenesis in vitro and in vivo are unique. Using a metabolomics approach, we discovered that both oxidative stress and GSH biosynthesis increase during osteoclastogenesis. Inhibiting GSH biosynthesis in vitro via the pharmacological or genetic inhibition of glutamate cysteine ligase (GCLC) prevented osteoclast differentiation. Conversely, the genetic ablation of GCLC in myeloid cells using *LysMCre* resulted in a decrease in bone mass in both male and female mice. The decreased bone mass of the *LysMCre;Gclc^fl/fl^* mice was attributed to increased osteoclast numbers and elevated bone resorption. Collectively, our data provide strong genetic evidence that GSH biosynthesis is essential for the regulation of osteoclast differentiation and bone resorption in mice. Moreover, these findings highlight the necessity of complementing in vitro studies with in vivo genetic studies.

## 1. Introduction

Osteoclasts are specialized multinucleated cells that secrete acid and collagenolytic enzymes that serve to resorb calcified bone matrix [1]. Osteoclasts differentiate from myeloid progenitor cells in response to macrophage colony-stimulating factor (M-CSF) and receptor activator of nuclear factor-kB ligand (RANKL) [1,2,3]. M-CSF regulates the proliferation and survival of osteoclast precursors, whereas RANKL induces the expression of critical transcription factors like cFos, and NFATc1, that regulate genes involved in osteoclast differentiation, including *Acp5* (TRAP), *Dc-Stamp*, *Atp6bd0d2*, *Itgb3*, and *Ctsk* [4,5,6,7,8,9]. During differentiation, mononuclear osteoclasts undergo fusion, polarize, and eventually form the F-actin sealing zone and ruffled membrane as they begin to secrete acids and enzymes into the resorbing lacunae [1,2,3,10]. Osteoclastic bone resorption is essential for bone health and maintaining bone homeostasis [11,12,13,14]. Increased osteoclast differentiation and bone resorption underlies many pathological bone and joint diseases, including postmenopausal osteoporosis, Paget’s bone disease, periprosthetic osteolysis, rheumatoid arthritis, and osteolytic bone metastasis [15,16,17,18,19].

Osteoclast differentiation is associated with an increase in the production of ROS, most notably superoxide anions (O_2_^−^) and hydrogen peroxide (H_2_O_2_), which can enhance osteoclast differentiation and bone resorption in vivo [20,21,22]. Both M-CSF and RANKL directly stimulate the production of ROS that are essential for osteoclast survival, proliferation, and differentiation [23,24,25]. ROS serve as secondary messengers, promoting the activation of PGC1b and NFATc1 to facilitate mitochondrial biogenesis and osteoclast differentiation [26,27]. Experiments using various antioxidants have confirmed that osteoclastogenesis is intricately tied to ROS production [25,28,29,30]. Along these lines, studies of mice have validated the importance of ROS. For example, increasing ROS levels through the genetic ablation of antioxidant proteins like nuclear factor erythroid 2 (NRF2) promotes osteoclast differentiation and bone resorption in vivo [31,32,33,34]. Likewise, the genetic deletion of the ROS-generating NADPH oxidases (NOX) NOX2 and NOX4 results in decreased osteoclast numbers in vivo [26,35]. On the other hand, reducing ROS levels via systemic administration of antioxidants like NAC or ascorbate or via genetic expression of the mitoCAT transgene in osteoclast lineage cells reduces osteoclast numbers and increases bone mass [28,29]. Despite this knowledge, the mechanisms governing ROS levels in osteoclasts remain under-studied.

**Figure 1:**
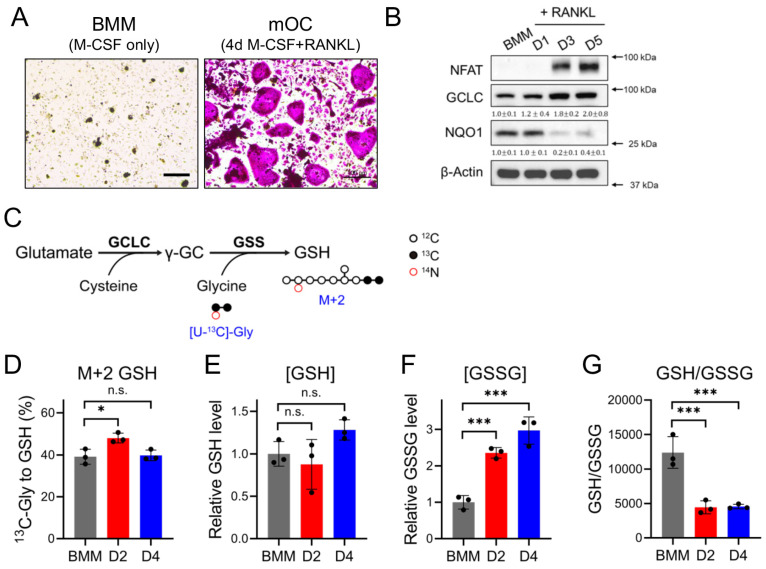
Transient increase in GSH biosynthesis during osteoclastogenesis. (**A**) Representative images of TRAP-stained bone marrow macrophages (BMMs) and mature osteoclasts (mOCs). Scale bar: 100 μm. (**B**) Western blot analysis of NFAT, GCLC, and NQO1 throughout osteoclast differentiation (*n* = 3). All proteins were normalized to β-actin. (**C**) Schematic showing GSH biosynthesis pathway and [U-^13^C]-glycine tracing. Black, filled circles indicate ^13^C; black, open circles denote ^12^C; red, open circles denote ^14^N. γ-GC, γ-glutamylcysteine; GSS, GSH synthetase. (**D**) Fractional contribution of [U-^13^C]-glycine to GSH (*n* = 3). (**E**,**F**) Relative intracellular GSH (**E**) or GSSG (**F**) in BMMs or following 2- or 4-day RANKL stimulation (*n* = 3). (**G**) GSH/GSSG ratio as measured via mass spectrometry (*n* = 3). Data are shown as means ± SD of 1-way ANOVA (**D**–**G**). * *p* < 0.05; *** *p* < 0.001; n.s., not significant.

O_2_^−^ can be converted into H_2_O_2_ by superoxide dismutase (SOD). H_2_O_2_ is detoxified to nonreactive H_2_O by several enzymes, including the GSH peroxidases (GPXs), which use GSH as the reducing agent [36]. GSH is a tripeptide composed of glutamate, cysteine, and glycine [37,38,39]. GSH is synthesized by the enzymes glutamate–cysteine ligase (GCL) and GSH synthase (GSS). GCL, a heterodimer composed of catalytic (GCLC) and modifier (GCLM) subunits, conjugates glutamate and cysteine to form γ-glutamylcysteine (γ-GC). GSS then adds a glycine to form GSH (Figure 1C) [40,41,42,43]. The roles of GCLC and GSH in osteoclastogenesis are controversial. Several research groups have demonstrated that the addition of exogenous GSH reduces osteoclast differentiation in vitro and that GSH supplementation prevents LPS-induced bone loss in vivo [25,30]. Along these lines, knockout of the deubiquitinating enzyme BAP1 resulted in metabolic changes, including increased levels of GSH, resulting in deficient osteoclast differentiation and bone resorption [44]. However, another study found that exogenous GSH potentiated osteoclast differentiation in vitro [45]. In this same study, reducing GSH concentrations by inhibiting GCLC using buthionine sulfoximine (BSO) reduced osteoclast differentiation both in vitro and in vivo [45]. However, Lean and colleagues found that BSO treatment increased osteoclast numbers both in vitro and in vivo and reduced bone mass [28]. These discrepancies highlight the need for rigorous genetic research into the roles of GCLC and de novo GSH biosynthesis during osteoclastogenesis and bone resorption.

In this study, we tested the hypothesis that GSH biosynthesis is essential for osteoclastogenesis. We used diverse approaches to inhibit GCLC in myeloid lineage cells. The effects of GCLC inhibition were evaluated using a combination of cell-based and metabolomic approaches in vitro and comprehensive bone phenotyping and histological characterization to comprehensively investigate how GSH biosynthesis regulates osteoclast differentiation and bone resorption. These data demonstrate that differentiating osteoclasts transiently increase GCLC expression and GSH biosynthesis. Pharmacological inhibition and genetic ablation of GCLC completely prevented osteoclast differentiation in vitro. Conversely, genetically ablating GCLC in myeloid cells accelerated osteoclast differentiation and increased bone resorption in vivo. Collectively, these data demonstrate that GCLC and GSH have unique requirements during osteoclastogenesis in vitro and in vivo.

## 2. Materials and Methods

### 2.1. Mouse Strains

*Gclc^fl^* mouse strain has been previously described [46]. *C57Bl/6 J*, *Rosa26^Cas9^* (Strain # 026179) and *LysMCre* (Strain #004781) strains were obtained from the Jackson Laboratory. All mice were housed at 23 °C and maintained on a 12 h light/dark cycle with free access to water and PicoLab Rodent Diet 290. All animal use procedures were approved by the Institutional Animal Care and Use Committee (IACUC) at the University of Texas Southwestern Medical Center of Dallas, Texas. For the animal study, 28 two-month-old mice were used in total (wild-type group: 8 male mice and 6 female mice; *LysM;Gclc^fl/fl^* group: 8 male mice and 6 female mice). All experimental evaluations were performed in a blinded and coded manner. The test for normality of data distribution was performed using DATAtab.

### 2.2. Micro-Computed Tomography (μCT)

Micro-computed tomography (μCT) (μCT45, Scanco Medical AG, Wangen-Brüttisellen, Switzerland, set at 55 kVp and 145 μA, Voxel size: 4.9 μm) was used for three-dimensional reconstruction and quantification of trabecular and cortical bone parameters. Specifically, femora were isolated, and all muscle and other tissue was removed. Femora were washed in PBS and fixed in 10% neutral buffered formalin for 48 h, immobilized using gauze, and scanned. Bone parameters were quantified from 400 slices directly underneath the growth plate with the threshold set at 300 to 1000.

### 2.3. Histology

Static histomorphometry procedures were performed on femora fixed in 10% neutral buffered formalin for 48 h at 4 °C. Femora were then decalcified by soaking them in 14% EDTA for two weeks, with daily changes. Decalcified femora were then embedded in paraffin and sectioned. Subsequently, 5 μm thick sections were stained with either hematoxylin and eosin (H&E) or tartrate-resistant acid phosphatase (TRAP) using standard protocols. For osteocalcin (OCN) immunostaining, antigen retrieval was performed by incubating tissue sections in 10 μg/mL of proteinase K (Sigma, St. Louis, MO, USA, Cat#1.24568) for 10 min followed by a 10 min incubation in 3% H_2_O_2_ (*v*/*v* in water). For CTSK/8-OHdG co-immunostaining, antigens were retrieved by boiling the slides in citric-acid-based antigen unmasking solution (Vector Laboratories, Newark, CA, USA, Cat#H-3300-250, 1:100 dilution) for 10 min. For all immunostaining experiments, sections were blocked in 0.06% normal goat serum (Vector Laboratories, Newark, CA, USA, Cat#S-1000-20, *v*/*v* in PBST) at room temperature for 30 min and then incubated overnight at 4 °C with anti-OCN (Millipore, Middlesex County, MA, USA, Cat#AB10911, 1:200 dilution), anti-8-OHdG (Abcam, Cambridge, UK, Cat#ab48508, 1:200 dilution), or anti-CTSK (Proteintech, Rosemont, IL, USA Cat#11239-1-AP, 1:200 dilution) and then incubated again with Alexa Fluor 568 goat anti-rabbit (Invitrogen, Waltham, MA, USA Cat#A-11011, 1:200 dilution) or Alexa Fluor 488 goat anti-mouse (Invitrogen, Waltham, MA, USA Cat#A-11001, 1:200 dilution) secondary antibody at room temperature for 30 min and mounted with antifade mounting medium with DAPI (Vector Laboratories, Newark, CA, USA, Cat#H-2000-10). Static histomorphometry was quantified using Image J Version 1.54 (https://imagej.net) (accessed on 1 July 2024).

### 2.4. Serum Analysis

Mice were fasted for 3 h prior to blood collection via cardiac puncture. Blood samples were stored at room temperature for 2 h to allow clotting. Samples were then centrifuged for 10 min at approximately 1000× *g*. Serum samples were then stored at −80 °C for later analyses. The amounts of P1NP, CTX-I, and TRAP5b in the serum were measured using ELISA for either P1NP (Immunodiagnostic Systems, East Boldon, UK, Cat#AC-33F1), CTX-I (Immunodiagnostic Systems, East Boldon, UK, Cat#AC-02F1), or TRAP5b (Immunodiagnostic Systems, East Boldon, UK, Cat#SB-TR103).

### 2.5. Monocyte/Macrophage Isolation and Osteoclast Culture

Primary bone marrow macrophages (BMMs) were isolated from age- and sex-matched mice. The femora and tibiae were isolated, and all connective tissue was rapidly removed. The epiphyses were cut off, and the bone marrow was collected via centrifugation for 5 s at approximately 2000× *g*. Red blood cells were lysed for 20 s in red blood cell lysis buffer (Roche, Basel, Switzerland, Cat#11814389001). Then, the bone marrow cells were cultured in an untreated polystyrene dish (Falcon, Flowery Branch, GA, USA, Cat#351029) containing expansion media (α-MEM media containing 10% FBS, 100 U/mL of penicillin G, 100 μg/mL of streptomycin, and 25 ng/mL of M-CSF (R&D Systems, Minneapolis, MN, USA, Cat#216-MC)) under a humidified atmosphere of 5% CO_2_ at 37 °C. After 2 days, cells were trypsinized, counted, and seeded at a density of 4.5 × 10^4^/cm^2^ and then cultured in osteoclast differentiation media (α-MEM media supplemented with 25 ng/mL of M-CSF and 40 ng/mL of RANKL (R&D Systems, Minneapolis, MN, USA, Cat#462-TEC)) for 4–5 days. Culture media were changed every two days. BSO (Sigma, St. Louis, MO, USA, Cat#B2515, 250 μM) or vehicle (DMSO) was included in the culture media, as indicated in the figure legend. TRAP staining was performed with an acid phosphatase leukocyte diagnostic kit in accordance with the manufacturer’s instructions.

### 2.6. CRISPR/Cas9-Mediated Gene KO

**Figure 2:**
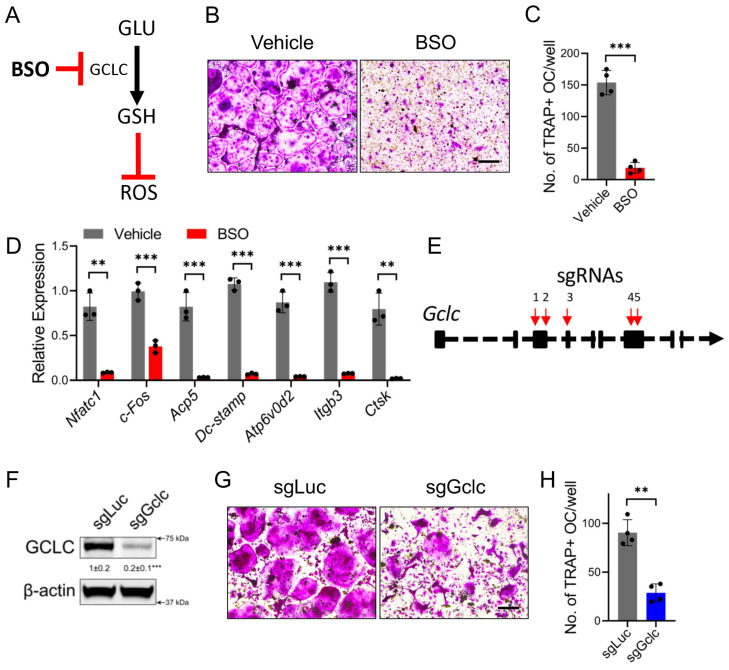
GCLC activity is required for osteoclast differentiation in vitro. (**A**) Schematic showing BSO inhibition of GCLC and GSH pathways. (**B**) Representative images of TRAP-staining showing the effect of BSO on osteoclast differentiation (*n* = 3). Scale bar: 100 μm. Images in (**B**) quantified in (**C**) (*n* = 3). (**D**) Effect of BSO treatment on the expression of osteoclast marker genes (*n* = 3). (**E**) Schematic depicting sgRNA targeting *Gclc* exons. (**F**) Results of Western blot analysis of GCLC and β-actin in *Gclc* (sgGclc)-targeted or control (sgLuc) BMMs (*n* = 3). (**G**) Representative images of TRAP-staining showing osteoclast differentiation of *Gclc* (sgGclc)-targeted or control (sgLuc) BMM cultures (*n* = 3). Scale bar: 100 μm. Images in (**G**) quantified in (**H**) (*n* = 3). Data are shown as means ± SD. Results of 2-tailed Student’s unpaired *t* test (**C**,**D**,**H**). ** *p* < 0.01, *** *p* < 0.001.

Lentiviral vectors expressing single-guide RNAs (sgRNAs) targeting either *Gclc* or Luciferase and mCherry were cloned into the LentiGuide-Puro plasmid according to a previously published protocol [47]. Then, the primary BMM cells isolated from *Rosa26^Cas9^* mice were infected with sgRNA-carrying lentivirus. The LentiGuide-Puro plasmid was a gift from Feng Zhang (Massachusetts Institute of Technology, Cambridge, MA, USA) (Addgene, Watertown, MA, USA, Cat#52963). Sequences of each sgRNA protospacer are shown in Table 1. The effectiveness of sgRNA sequences was verified via Western blot (Figure 2F) and in [46].

### 2.7. Virus Production

For virus creation, the indicated vector was co-transfected with the plasmids pMD2.g and psPax2 into HEK293 cells. After 48 h, virus-containing media were collected, filtered through a 0.45 μm filter, and then stored at −80 °C for later use. For infection, BMM cells were infected at 50% confluency for 24 h and then recovered in regular media for another 24 h. Then, selection was performed using puromycin (5 μg/mL) for 48 h.

### 2.8. Analysis of GSH Using LC-MS/MS

Glutathione was analyzed using LC-MS as we have previously described, with some minor differences [46]. Briefly, BMMs were induced to undergo osteoclastogenesis for 0, 2, or 4 days and then cultured in the presence of 1.33 mM [U-^13^C] glycine for 14 h. Following this incubation period, metabolites were extracted two times using 250 μL of ice-cold methanol containing 10 mM N-Ethylmaleimide on dry ice. The extracts were then sonicated for 1 min and then centrifuged at 13,000× *g* for 1 h at 4 °C. The supernatant was then completely dried using N_2_ gas, and the dried residue was redissolved in 50 μL of H_2_O and transferred to LC-MS vials for analysis, as we previously described [46].

### 2.9. RNA Isolation and qPCR

Total RNA from cells was extracted using TRIzol reagent (ThermoFisher, Waltham, MA, USA, Cat#15596018). A total of 500 ng of total RNA was used for cDNA synthesis using the iScript cDNA synthesis kit (Bio-Rad, Hercules, CA, USA, Cat#1708841). qPCR was performed in technical and biological triplicates using an ABI Quant Studio 3, using SYBR green chemistry (Bio-Rad, Hercules, CA, USA, Cat#1725275). Gene expression was normalized to *Actb* mRNA, and the relative expression was calculated using the 2^−(ΔΔCt)^ method. Primers were used at 0.1 μM, and their sequences are listed in Table 2 and [48].

### 2.10. Western Blotting

BMMs were lysed in 120 µL RIPA buffer (50 mM Tris–HCl pH 7.4, 150 mM NaCl, 1% NP-40, 0.5% sodium deoxycholate, and 0.1% SDS) supplemented with protease (Roche, Minneapolis, MN, USA, Cat#11697498001) and phosphatase inhibitors (Roche, Cat#04906837001). Protein fractions were isolated via centrifugation at ~15,000× *g* at 4 °C for 10 min. Protein concentration was determined empirically using the BCA method. Protein samples were then mixed with loading buffer and boiled for 10 min. A total of 20 μg of total protein was resolved using a 4–15% polyacrylamide gel and then transferred onto a polyvinylidene difluoride (PVDF) membrane. The membranes were incubated in blocking buffer (5% milk in TBST (TBS, 0.1% Tween 20)) for 1 h at room temperature with gentle agitation. Membranes were then incubated with primary antibodies overnight in blocking buffer at 4 °C. The following primary antibodies were used in this study: anti-GCLC (Abcam, Cambridge, UK, Cat#ab53179, 1:1000 dilution), anti-NQO1 (Cell Signaling Technology, Danvers, MA, USA, Cat#62262, 1:1000 dilution), anti-NFATc1 (BD Biosciences, Franklin Lakes, NJ, USA, Cat#556602, 1:250 dilution), anti-Prdx1-SO_3_ (Abcam, Cambridge, UK, Cat#ab16830, 1:1000 dilution), and anti-β-actin (Cell Signaling Technology, Danvers, MA, USA, Cat#4970, 1:1000 dilution). Membranes were then washed 3 times using TBST and further incubated with HRP-linked anti-rabbit (Cell Signaling Technology, Danvers, MA, USA, Cat#7074, 1:2000 dilution) or HRP-linked anti-mouse (Cell Signaling Technology, Danvers, MA, USA, Cat#7076, 1:2000 dilution) secondary antibodies in 5% milk for 1 h at room temperature. All blots were developed using either the Clarity ECL substrate (Bio-Rad, Hercules, CA, USA, Cat#1705060) or the Super Signal West Femto substrate (ThermoFisher, Waltham, MA, USA, Cat#PI34095).

### 2.11. Statistical Analysis

We used GraphPad Prism 10 to perform all statistical analyses. All data in the graphs are presented as means ± standard deviations. In BMM studies, we used either an unpaired 2-tailed Student’s *t* test or one-way ANOVA to determine statistical significance. For animal studies, we used a paired 2-tailed Student’s *t* test to determine statistical significance, comparing paired littermate controls. We considered a *p* value < 0.05 statistically significant. All experiments were performed with a minimum of 3 or more biological replicates.

## 3. Results

### 3.1. GSH Biosynthesis Increases During Osteoclastogenesis

Osteoclast differentiation is associated with increased production of reactive oxygen species (ROS) [20,22]. Increased ROS production is attributed to increased mitochondrial activity and reduced expression of antioxidant proteins. Indeed, the levels of several antioxidant proteins, including nuclear factor erythroid 2-related factor 2 (NRF2) and NAD(P)H quinone dehydrogenase 1 (NQO1), decline during osteoclast differentiation [22] (Figure 1A,B). By comparison, GCLC expression increases during osteoclast differentiation (Figure 1B). Stable isotope tracing using uniformly ^13^C labeled glycine ([U-^13^C]-glycine) (Figure 1C) revealed that there was robust de novo GSH biosynthesis in the unstimulated BMMs, as ~40% of the GSH pool was M + 2 labeled over the course of 14 h (Figure 1D). Moreover, there was a transient increase in M + 2-labeled GSH after 2 days of RANKL treatment, a finding consistent with increased GCLC expression (Figure 1D). Interestingly, the total GSH levels were unchanged, whereas the levels of oxidized GSH (GSSG) substantially increased during differentiation (Figure 1E,F). Moreover, the GSH/GSSG ratio was significantly reduced, which is consistent with elevated ROS levels and increased oxidative stress (Figure 1G). These data suggest that GSH biosynthesis increases during early osteoclastogenesis—likely to manage differentiation-associated ROS production.

To determine the necessity of GSH biosynthesis for osteoclastogenesis, we inhibited GSH biosynthesis first using buthionine sulfoximine (BSO), a chemical inhibitor of GCLC (Figure 2A). TRAP staining revealed that BSO inhibited the formation of TRAP+ multinucleated osteoclasts (Figure 2B,C). Consistent with the TRAP-staining results, BSO inhibited the induction of many markers of osteoclast differentiation, including early regulatory transcription factors (e.g., *Nfatc1* and *cFos*), marker genes (e.g., *Acp5*), fusion genes (e.g., *DC-stamp*, *Atp6v0d2*, and *Itgb3*), and mature osteoclast marker genes (e.g., *Ctsk*) (Figure 2D). To ensure the specificity of BSO treatment, we next knocked out *Gclc* in BMMs using CRSIPR/Cas9 targeting (Figure 2E). Western blot analysis confirmed that this approach reduced GCLC levels by ~75% in the BMMs (Figure 2F). Consistent with the BSO-mediated GCLC inhibition, the sgGclc BMMs formed significantly fewer osteoclasts compared to the BMMs infected with sgRNAs targeting *mCherry* and *Luciferase* (denoted as sgLuc) (Figure 2G,H). Together, these data indicate that GCLC-dependent GSH biosynthesis is essential for osteoclastogenesis in vitro.

### 3.2. GSH Biosynthesis Limits Osteoclast Differentiation In Vivo

**Figure 3:**
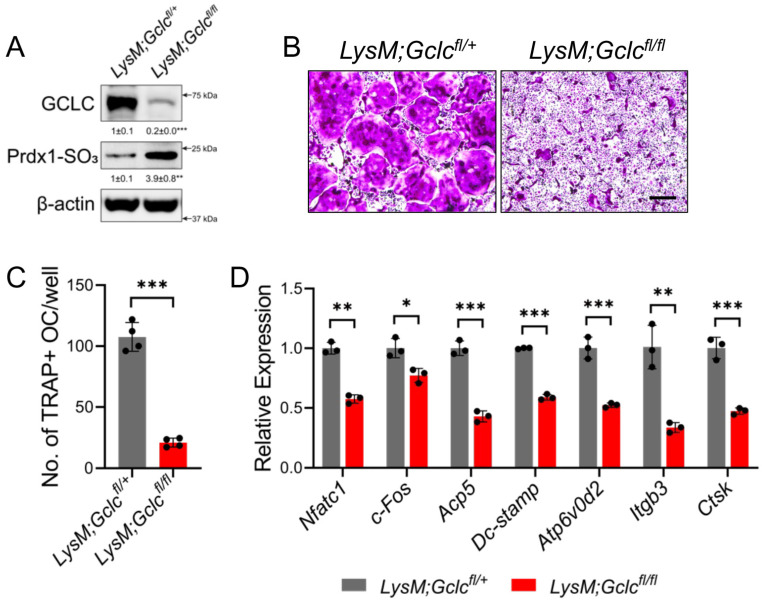
*LysMCre;Gclc^fl/fl^* BMMs have defective osteoclastogenesis in vitro. (**A**) Western blot analysis of GCLC, Prdx1-SO_3_, and β-actin in BMMs isolated from *LysMCre;Gclc^fl/+^* (wild type) or *LysMCre;Gclc^fl/fl^* mice (*n* = 3). All are proteins normalized to β-actin. (**B**) Representative images of TRAP-stained osteoclasts in BMM cultures from *LysMCre;Gclc^fl/+^* or *LysMCre;Gclc^fl/fl^* mice (*n* = 4). Scale bar: 100 μm. Images in (**B**) quantified in (**C**) (*n* = 4). (**D**) qPCR analysis showing reduced induction of osteoclast marker genes in *LysMCre;Gclc^fl/fl^* BMM cultures induced with RANKL for 4 days (*n* = 3). Data are shown as means ± SD of 2-tailed Student’s unpaired *t* test (**C**,**D**). * *p* < 0.05, ** *p* < 0.01, *** *p* < 0.001.

**Figure 4:**
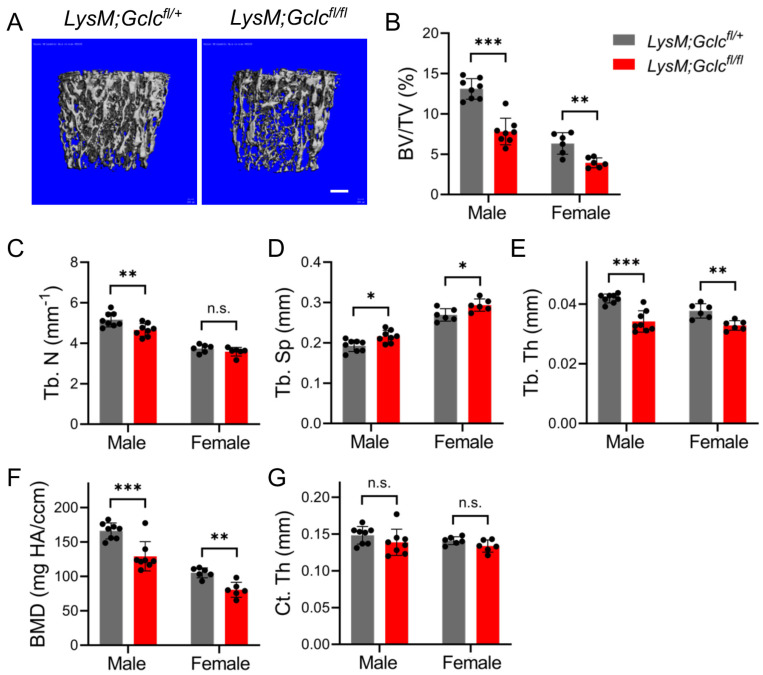
*LysMCre;Gclc^fl/fl^* mice have reduced bone mass in vivo. (**A**) Representative µCT images of the trabecular bone in the distal femur of 2-month-old *LysMCre;Gclc^fl/+^* (wild type) or *LysMCre;Gclc^fl/fl^* male mice. Scale bar: 200 μm. (**B**–**G**) Quantification of bone parameters determined by µCT: (**B**) BV/TV—bone volume/tissue volume; (**C**) Tb.N (mm^−1^)—trabecular number; (**D**) Tb.Sp (mm)—trabecular separation; (**E**) Tb.Th (mm)—trabecular thickness; (**F**) BMD (mg HA/ccm)—bone mineral density; (**G**) Ct.Th (mm)—cortical thickness. (Male: *n* = 8; Female: *n* = 6). Data are shown as means ± SD of 2-tailed Student’s paired *t* test comparing *LysMCre;Gclc^fl/+^* to *LysMCre;Gclc^fl/fl^* for males and females, respectively (**B**–**G**). * *p* < 0.05; ** *p*< 0.01; *** *p* < 0.001; n.s., not significant.

We next sought to determine the physiological impact of the loss of GCLC in osteoclasts in vivo. To test this, we generated *LysMCre;Gclc^fl/fl^* mice in which a floxed allele of *Gclc* (*Gclc^fl^*) was conditionally ablated in myeloid-lineage osteoclast-precursors. GCLC protein was efficiently depleted in *LysMCre;Gclc^fl/fl^* BMMs (Figure 3A). No obvious appearance or behavior changes were observed between either the male or female wild-type and *LysMCre;Gclc^fl/fl^* mice. Consistent with reduced GSH biosynthesis and increased ROS levels, we observed the oxidation of peroxiredoxin-1 (PRDX1) cysteine residues (PRDX1-SO3) increased ~3.8 fold in the *LysMCre;Gclc^fl/fl^* BMMs (Figure 3A). Next, we performed differentiation assays. The *LysMCre;Gclc^fl/fl^* BMMs formed significantly fewer TRAP+ multinucleated osteoclasts in vitro compared to the BMMs isolated from wild-type littermates (Figure 3B,C). Loss of *Gclc* appeared to affect early osteoclast differentiation, as the levels of the upstream transcription factors *cFos* and *Nfatc1* were significantly reduced in *LysMCre;Gclc^fl/fl^* osteoclasts (Figure 3D). Likewise, *Gclc* ablation inhibited the induction of osteoclast differentiation, fusion, and maturation genes (Figure 3D). Next, we evaluated bone phenotypes in the *LysMCre;Gclc^fl/fl^* mice. Interestingly, µCT analyses revealed that 2-month-old *LysMCre;Gclc^fl/fl^* mice had significantly lower trabecular bone volume per tissue volume (BV/TV) compared to the wild-type littermate controls for both males and females (Figure 4A,B). Analysis of the µCT scans revealed the trabecular number (Tb.N) was significantly lower in *LysMCre;Gclc^fl/fl^* males but not females (Figure 4C). Trabecular separation (Tb.Sp) was significantly increased, whereas trabecular thickness (Tb.Th) and bone mineral density (BMD) were significantly decreased, in both male and female *LysMCre;Gclc^fl/fl^* mice (Figure 4D,F). Cortical parameters were unaffected in both male and female mice at 4 months of age compared to the wild-type littermate controls (Figure 4G). These data indicate that GCLC is required cell autonomously in myeloid progenitor to regulate bone mass in vivo.

**Figure 5:**
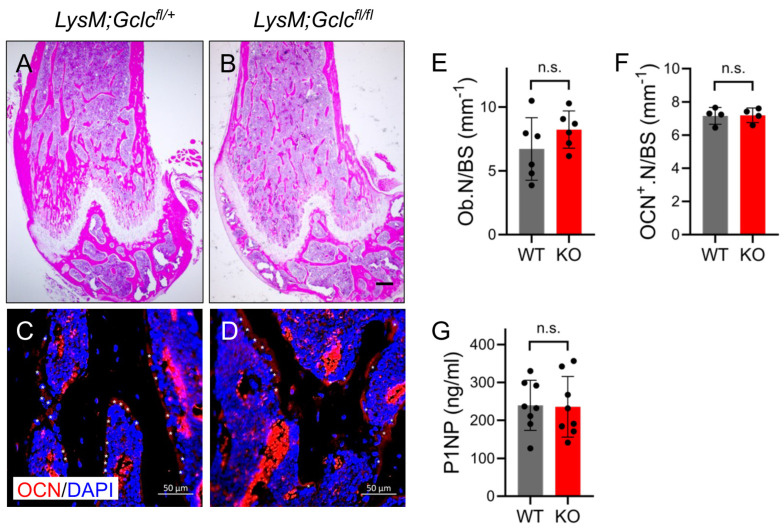
Reduction in bone mass is not due to reduced osteoblast numbers or activity. (**A**–**D**) H&E staining or (**C**,**D**) osteocalcin (OCN) immunostaining of the distal femur from 2-month-old *LysMCre;Gclc^fl/+^* (**A**,**C**) or *LysMCre;Gclc^fl/fl^* (**B**,**D**) male mice. Scale bar in (**A**,**B**) is 100 μm. Scale bar in (**C**,**D**) is 50 μm. (**E**) Osteoblast number per bone surface (Ob.N/BS) quantified from H&E staining (*n* = 6). (**F**) OCN^+^ osteoblast number per bone surface (OCN^+^.N/BS) quantified from OCN immunostaining (*n* = 4). (**G**) Serum levels of P1NP measured via ELISA (*n* = 8). Data are shown as means ± SD of 2-tailed Student’s paired *t* test (**E**–**G**). n.s., not significant.

**Figure 6:**
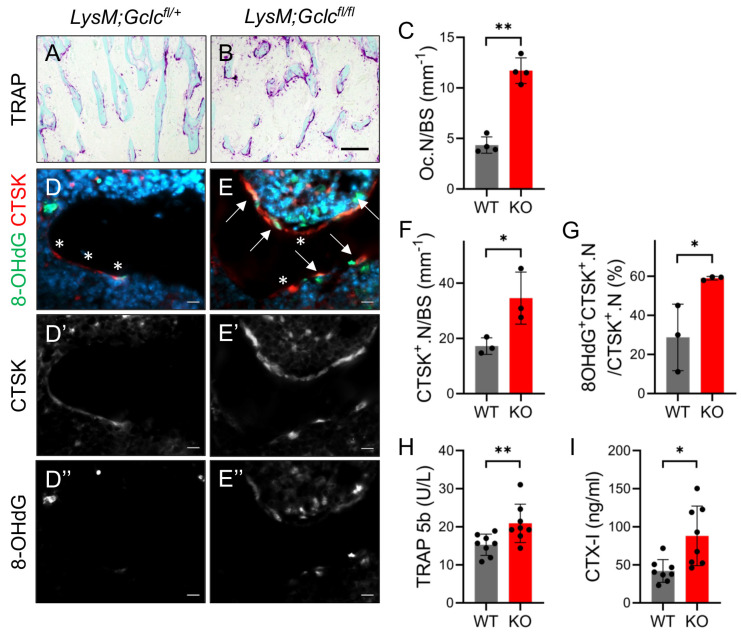
*LysMCre;Gclc^fl/fl^* mice have increased osteoclast numbers and activity. (**A**,**B**) Representative TRAP staining performed on distal femur of 2-month-old *LysMCre;Gclc^fl/+^* or *LysMCre;Gclc^fl/fl^* male mice. Scale bar denotes 100 μm. (**C**) Osteoclast number per bone surface (Oc.N/BS) quantified from TRAP staining (*n* = 4). (**D**,**E**) Representative Cathepsin K (CTSK) and 8-OHdG immunostaining performed on distal femur of 2-month-old *LysMCre;Gclc^fl/+^* or *LysMCre;Gclc^fl/fl^* male mice. Scale bar denotes 50 μm in (**D**,**D’**,**D”**,**E**,**E’**,**E”**). Single channel images of CTSK (**D’**,**E’**) or 8-OHdG (**D”**,**E”**) shown below. * denotes CTSK^+^ osteoclasts, and arrows denote CTSK/8-OHdG double-positive osteoclasts. (**F**) Quantification of CTSK^+^ osteoclast number per bone surface (CTSK^+^.N/BS) (*n* = 3). (**G**) Quantification of the percentage of osteoclasts that are 8-OHdG-positive (*n* = 3). (**H**,**I**) Serum levels of TRAP 5b (**H**) and CTX-I (**I**) measured via ELISA (*n* = 8). Data are shown as means ± SD of 2-tailed Student’s paired *t* test (**C**,**F**–**I**). * *p* < 0.05, ** *p* < 0.

To determine the cause of the decreased bone mass in the *LysMCre;Gclc^fl/fl^* mice, we performed histological analyses. H&E staining confirmed there was a reduction in trabecular bone mass in the *LysMCre;Gclc^fl/fl^* mice compared to that of the wild-type littermate controls (Figure 5A,B). The decrease in bone mass in the *LysMCre;Gclc^fl/fl^* mice was not due to changes in the number of osteoblasts, as determined via both static histomorphometry and osteocalcin (OCN) immunofluorescence staining (Figure 5C–F). Likewise, serum N-terminal pro-peptide of type 1 collagen (P1NP), a bone formation marker, was not affected in the *LysMCre;Gclc^fl/fl^* mice (Figure 5G). These results indicate that the decreased bone mass in *LysMCre;Gclc^fl/fl^* mice was not caused by changes in osteoblast differentiation or bone formation. Conversely, the *LysMCre;Gclc^fl/fl^* mice had significantly more TRAP^+^ osteoclasts per bone surface (Oc.N/BS) (Figure 6A–C). Likewise, the *LysMCre;Gclc^fl/fl^* mice had significantly more CTSK^+^ mature osteoclasts per bone surface (CTSK^+^.N/BS) (Figure 6D–F). Co-immunostaining with 8-OHdG, a marker of DNA oxidation, revealed that almost 60% of the CTSK-positive osteoclasts in the *LysMCre;Gclc^fl/fl^* mice were also 8-OHdG^+^, compared to only ~25% of wild-type osteoclasts (Figure 6D–G). This indicates *LysMCre;Gclc^fl/fl^* osteoclasts had elevated ROS levels in vivo. Serum analyses revealed that the *LysMCre;Gclc^fl/fl^* mice had significantly elevated serum TRAP5B and C-terminal telopeptide of collagen (CTX-1) levels consistent with increased osteoclast activity (Figure 6H,I). Thus, GCLC functions cell-autonomously in myeloid cells to restrain osteoclastogenesis and bone resorption in vivo.

## 4. Discussion

Here, we describe contradictory requirements for GCLC and GSH biosynthesis during osteoclastogenesis in vitro and in vivo. In vitro, inhibiting GCLC activity prevented osteoclast differentiation, suggesting GCLC activity is required for osteoclastogenesis. On the other hand, the genetic ablation of *Gclc* in myeloid cells in vivo resulted in osteoclast expansion and accelerated bone resorption, suggesting GCLC serves to limit osteoclastogenesis and bone resorption. This is consistent with recent genetic studies demonstrating that an increase in GSH levels is associated with reduced osteoclast function and bone resorption in vivo [44].

RANKL stimulates ROS production, which is critical for MAPK activation and the induction of pro-osteoclastogenic transcriptional programs [23]. Increased ROS likely follows increased protein synthesis and mitochondrial biogenesis coupled with reduced expression of several antioxidant proteins during osteoclast differentiation [22]. Several studies have shown that reducing ROS levels using supraphysiological doses of GSH (20 mM) or NAC (10–30 mM) inhibits osteoclast differentiation and activity both in vitro and in vivo [23,25,30,34,49,50]. Likewise, reducing ROS levels by inhibiting the NADPH oxidase or expressing catalase prevents osteoclast differentiation in vitro [23]. Interestingly, some groups found that lower, more physiological doses of GSH (1–5 mM) or NAC (1–5 mM) enhanced osteoclast formation and bone resorption [45,51,52]. Moreover, low doses of BSO (≤10 μM) were shown by one group to promote osteoclastogenesis in vitro while higher doses (10–300 μM BSO) were inhibitory [45,50,51]. One could conclude from these studies that ROS have threshold effects in osteoclasts where high and low ROS concentrations are not consistent with osteoclastogenesis. If ROS levels are too low, osteoclast transcriptional programs will not be sufficiently activated and osteoclastogenesis will fail. Conversely, ROS levels above the threshold, due to GCLC knockout or inhibition (e.g., BSO) in vitro [45,50,51] (Figure 1, Figure 2 and Figure 3), are detrimental and inhibit osteoclastogenesis. Between these two extremes, ROS have pro-osteoclastogenic effects, wherein an increase in ROS doses enhances osteoclastogenesis-associated transcriptional programs and bone resorption. This is most apparent under the physiological conditions of aging/estrogen deficiency [53,54,55] or in the *LysMCre;Gclc^fl/fl^* mice (Figure 4, Figure 5 and Figure 6). Here, ROS levels were elevated but likely beneath the upper threshold preventing oxidative damage and promoting osteoclastogenesis.

It is unclear why GCLC ablation has unique effects on osteoclastogenesis in vitro and in vivo. We postulate that this could result from differences in oxygen tension or nutrient availability. The bone marrow microenvironment is considered hypoxic at around 0.6–2.8% oxygen tension [56]. By comparison, cell culturing is typically performed at atmospheric oxygen tension (i.e., 20% oxygen) [57]. There is a linear relationship between mitochondrial ROS production and oxygen tension [58,59,60,61]. The higher oxygen tension in vitro likely potentiates mitochondrial activity and enhances basal ROS levels. This is predicted to create a greater need for GCLC and GSH to mitigate ROS levels and maintain osteoclast potential in vitro. Here, when GCLC is inhibited, ROS levels are predicted to become too high, which would inhibit osteoclast differentiation. By comparison, the hypoxic bone marrow environment likely reduces mitochondrial OXPHOS levels compared to those in vitro. We predict this results in lower ROS levels, a state we predict requires less GCLC and GSH biosynthesis. In this scenario, when GCLC is ablated, ROS levels are predicted to rise to a level that enhances osteoclastogenesis but remains below the damaging threshold.

Nutrient availability may also influence the necessity for GCLC and GSH biosynthesis. The in vitro environment is nutrient-rich when compared to the bone marrow microenvironment. In cultures, osteoclasts have an abundant supply of glucose, fatty acids, and amino acids that are essential for osteoclast differentiation [48,62,63,64,65,66]. While the concentration of glucose in our culture media is similar to that in plasma (e.g., 5 mM), glutamine and glutamate are present at 2 mM and 0.5 mM in culture media, which can be compared to concentrations of around 0.5 mM and 10 μM, respectively, in plasma [67,68]. GSH biosynthesis depends on an ample supply of glutamine, glutamate, glycine, and cysteine. Amino acids also promote protein synthesis, which is essential for osteoclastogenesis and is a major source of ROS via oxidative protein folding [69,70]. Moreover, glucose and fatty acids are preferred substrates for OXPHOS in osteoclasts, the primary source of ROS in cells [62,63,64]. It is logical to assume differences in nutrient availability may affect REDOX homeostasis by altering energetic metabolism and/or protein, nucleotide, and GSH synthesis. When nutrients are replete in vitro, osteoclasts likely increase GSH biosynthesis to offset the potential ROS burden that results from oxidative protein folding and OXPHOS [69,70,71]. GSH may thus become less important in vivo due to differences in nutrient and oxygen availability. Consistent with this idea, inhibiting glucose uptake or fatty acid oxidation inhibits osteoclast differentiation in vitro yet only results in mild sexually dimorphic osteoclast phenotypes in vivo [62,63,64]. Thus, caution should be taken when interpreting the results of in vitro experiments, especially with respect to experiments manipulating REDOX homeostasis and metabolism.

In summary, our data demonstrate that the depletion of GSH has distinct effects on osteoclastogenesis in vitro and in vivo. Thus, we conclude that the requirements for GSH biosynthesis during osteoclastogenesis in vitro and in vivo are unique. In cultures, GCLC enzymatic activity and GSH biosynthesis are essential to allow osteoclastogenesis to proceed. By comparison, inhibiting GSH synthesis in myeloid-lineage cells enhanced osteoclastogenesis and bone resorption, suggesting that GSH serves to limit osteoclastogenesis in vivo. This is strong genetic evidence that GCLC and GSH are essential regulators of osteoclast differentiation and bone resorption in mice. Furthermore, our study underscores the importance of using genetic mouse models to understand the role of metabolic pathways in osteoclasts.

## Figures and Tables

**Table 1:** List of sgRNA sequences used in this study.

Name	gRNA Sequence
SP742.GCLC.g4	TACATGATCGAAGGAACGCCNGG
SP742.GCLC.g19	TCAGACATCGTTCCTCCGTANGG
SP743.GCLC.g7	TGCTTGTTTATGGCTTCATCNGG
SP744.GCLC.g3	TAGTGGCCAGCTGATCATAANGG
SP744.GCLC.g4	TCTTGCCTCAGATATGCTGCNGG
SP498.mCherry.g17	CAAGTAGTCGGGGATGTCGGNGG
SP498.mCherry.g19	AGTAGTCGGGGATGTCGGCGNGG
SP499.LUC.g3	CAATTCTTTATGCCGGTGTTNGG
SP499.LUC.g4	GTGTTGGGCGCGTTATTTATNGG

**Table 2:** List of qPCR primers used in this study.

Gene Symbol	Forward	Reverse
*Nfatc1*	GGAGCGGAGAAACTTTGCG	GTGACACTAGGGGACACATAACT
*c-Fos*	CGGGTTTCAACGCCGACTA	TTGGCACTAGAGACGGACAGA
*Acp5*	CACTCCCACCCTGAGATTTGT	CATCGTCTGCACGGTTCTG
*Dc-stamp*	TACGTGGAGAGAAGCAAGGAA	ACACTGAGACGTGGTTTAGGAAT
*Itgb3*	CCACACGAGGCGTGAACTC	CTTCAGGTTACATCGGGGTGA
*Atp6v0d2*	CAGAGCTGTACTTCAATGTGGAC	AGGTCTCACACTGCACTAGGT
*Ctsk*	GAAGAAGACTCACCAGAAGCAG	TCCAGGTTATGGGCAGAGATT
*β-actin*	AGATGTGGATCAGCAAGCAG	GCGCAAGTTAGGTTTTGTCA

## Data Availability

Data are contained within the article.
